# Enzymatic extraction and response surface method optimization for efficient extraction of bioactive peptides and functional activities from *Leucopaxillus giganteus*

**DOI:** 10.3389/fnut.2026.1940279

**Published:** 2026-09-10

**Authors:** Cheng Zhang, Yan Guo, Shang Guo, Yanting Li, Lina Xu

**Affiliations:** Shanxi Institute for Functional Food, Shanxi Agricultural University, Taiyuan, China

**Keywords:** activity, antibacterial, enzymatic hydrolysis, *Leucopaxillus giganteus*, peptide

## Abstract

The *Leucopaxillus giganteus* is a typical high-protein wild edible fungus and is a high-quality natural plant protein source. In this study, the fruiting bodies of *Leucopaxillus giganteus* were adopted as experimental raw materials, and their intrinsic proteins were degraded via compound protease hydrolysis to produce fungus-derived bioactive peptides with antioxidant, antibacterial and anti-tumor properties. Single-factor tests were performed to determine the appropriate range of each hydrolysis factor, and Plackett–Burman design was adopted to screen three key variables significantly affecting peptide extraction efficiency, including hydrolysis temperature, time and pH. Box–Behnken response surface methodology was further applied to optimize the enzymatic hydrolysis process. The results indicated that the optimal compound protease ratio of alkaline protease to papain was 4:3. Under the optimal hydrolysis conditions (temperature 49 °C, enzyme dosage 5,870 U g^−1^, pH 7.1, solid–liquid ratio 1:25, hydrolysis time 5 h), the peptide extraction rate reached 28.93%. Subsequently, the activity of the peptide was determined. This peptide exhibited excellent DPPH scavenging ability and formed inhibition zones of 9.02 mm, 6.15 mm and 8.67 mm for *Escherichia coli, Bacillus subtilis and Staphylococcus aureus*, demonstrating good antibacterial activity. Moreover, the peptide could significantly inhibit the proliferation of Caco-2 colorectal cancer cells and HepG2 human liver cancer cells. In conclusion, the enzymatic hydrolysis process optimized by response surface methodology is simple and efficient, which lays a solid foundation for the development and comprehensive utilization of bioactive peptides derived from *Leucopaxillus giganteus*.

## 1 Introduction

Mushrooms are high-quality edible fungi. For a long time, due to their unique and delicate flavor, they have been used as flavoring ingredients in food, soups, and sauces (1, 2). In recent years, due to their rich nutritional value and pharmacological properties such as anti-tumor, anti-inflammatory, and antibacterial effects, mushrooms have received widespread attention (3–6) and have become an important raw material for the development of functional foods, medicines, and nutritional supplements.

*Leucopaxillus giganteus* is a common edible mushroom and belongs to the phyla Basidiomycete, order Agaricales, and family Tricholomataceae (7). *Leucopaxillus giganteus* belongs to the *Leucopaxillus genus*. As a medicinal and edible fungus, the *Leucopaxillus giganteus* is rich in various mineral elements such as calcium, iron, zinc, as well as multiple vitamins, polysaccharides, and other nutritional components (8). It can enhance the body’s resistance and cytotoxicity against pathogens and tumor cells (9), prevent cardiovascular diseases, lower cholesterol, and maintain normal metabolism. Current research on the nutritional components of the *Leucopaxillus giganteus* mainly focuses on polysaccharides (10). Studies have optimized the extraction process of polysaccharides from the *Leucopaxillus giganteus*, showing that these polysaccharides have a triple-helix structure, exhibit significant thermal stability, and possess good anticancer activity and antioxidant (11). Nevertheless, the high-value protein resource of *Leucopaxillus giganteus* has been largely neglected, and relevant research on its protein-derived bioactive peptides remains absent.

As a vital major nutrient, protein supplies the human body with indispensable amino acids and metabolic energy (12). Beyond fundamental nutritional functions, numerous food-derived proteins can exert additional physiological benefits by releasing bioactive peptides embedded within their amino acid sequences (13). Peptides from edible mushrooms are known for their easy absorption, high efficacy, minimal side effects, and high safety (14). Researchers have extracted peptides from various edible mushrooms, demonstrating their biological functions such as antioxidant properties, antibacterial effects, immune enhancement, and anticancer activities (15). The *Leucopaxillus giganteus* has a protein content as high as 41.84%, with a relatively high proportion of amino acid composition at around 33.31% (16), making it a potential source of high-quality protein. However, there is currently no reported research on this aspect. Peptide preparation methods can vary based on raw materials, objectives, and physicochemical properties, with enzymatic hydrolysis being a commonly used method due to its high extraction rate, low cost, ease of operation, and product safety (17). Researchers have utilized enzymatic hydrolysis to prepare mushroom peptides, comparing the enzymatic effects of alkaline protease, trypsin, papain, and a composite protease. On the other hand, protein solubility has been reported to correlate with enzyme type. Alkaline proteases can specifically degrade peptide bonds that contain tyrosine, glutamic acid or leucine on the carboxyl side, thereby increasing the solubility of proteins (18). Some bioactive peptides have been proven to have antibacterial effects, as well as humoral immunity and cell-mediated immune functions. Therefore, they are expected to be used as components in functional foods, nutritional supplements, and medicines. Their biological activity can help control and prevent diseases (19). However, there are currently no studies on the extraction and activity of the peptides from the *Leucopaxillus giganteus*.

In this study, the peptide of *Leucopaxillus giganteus* were extracted by the method of compound protease hydrolysis. The appropriate types and proportions of proteases (alkaline protease and papain) were screened. The peptide of *Leucopaxillus giganteus* were hydrolyzed and peptides were prepared. Through single-factor experiments, the effects of hydrolysis pH value, temperature, enzyme dosage and hydrolysis time on the peptide extraction rate were investigated. Subsequently, the Box–Behnken response surface experiment was used to optimize the hydrolysis process. Under the optimal conditions, the peptide content could reach 29.23%. The peptides obtained by this process exhibited significant antioxidant, anti-cancer and antibacterial activities. This process provides a theoretical basis for the development and utilization of peptides from *Leucopaxillus giganteus*.

## 2 Materials and methods

### 2.1 Chemicals and reagents

Alkaline protease (2000 U/mg, pH = 8, 50 °C), papain (80 U/mg, pH = 7, 50 °C) (both food-grade): purchased from Qingdao Haiweisen Technology Co., Ltd.; DMEM medium and Fetal bovine serum (Gibco, United States); Forskolin (1 mol/L, BR); Trichloroacetic acid (T104257, ≥ 99%), copper sulfate (C141239, ≥ 98%), potassium sodium tartrate (P639655 ≥ 99%), sodium carbonate (S141342, ≥ 99.5%), sodium hydroxide (S431791, ≥ 97%) (all analytical grade).

### 2.2 Plant materials

The fruiting bodies of *Leucopaxillus giganteus* used in this experiment were collected from the Lvliang Mountain area in Shanxi Province, China. The collection site is located at an altitude of 2249.90 meters, at 111°51′36 “east longitude and 38°46’12” north latitude. The fruiting bodies of *Leucopaxillus giganteus* were preserved at the Shanxi Institute for Functional Food, with the specimen number SXSYJBB1601. Freshly harvested *Leucopaxillus giganteus* fruiting bodies were oven-dried at 50 °C for 12 h, pulverized, and sieved using a 40-mesh screen. They were then sealed and stored at −20 °C.

### 2.3 Cell lines

Colorectal cancer cells (Caco-2) and liver cancer cells (HepG2) were purchased from Procell (Wuhan, China). Two types of cells were incubated at 37 °C in a sterile CO₂ incubator with DMEM medium, supplemented with 10% heat-inactivated fetal bovine serum (FBS) and 1% penicillin–streptomycin.

### 2.4 Preparation of peptides from *Leucopaxillus giganteus*

Freshly harvested *Leucopaxillus giganteus* fruiting bodies were oven-dried at 50 °C for 12 h, pulverized, and sieved using a 40-mesh screen. The mushroom powder was blended with distilled water at a solid–liquid ratio of 1:25 and pre-soaked for 30 min. Prior to enzymatic hydrolysis, ultrasonic pretreatment (200 W, 40 KHz) was performed on the suspension for 30 min; ultrasonic cavitation can break compact fungal cell walls, loosen protein cross-linking structures, and greatly enhance the exposure of protein substrates to proteases, which facilitates subsequent proteolytic cleavage. The pH of the ultrasound-treated suspension was adjusted to 7.2, then alkaline protease and papain were incorporated at predetermined ratios, followed by water-bath enzymatic hydrolysis at 50 °C. After hydrolysis, the mixture was immersed in boiling water for 15 min to fully terminate enzyme activity. Once cooled to room temperature, the sample was centrifuged at 4000 rpm for 20 min, and the supernatant was collected as crude polypeptide solution.

### 2.5 Determination of extraction rate of peptides from *Leucopaxillus giganteus*

The extraction rate of the peptide was determined by the Folin phenol method. 20% trichloroacetic acid was added to the enzymatic solution in an equal volume, and the mixture was left to stand for 10 min to allow the unenzymatically degraded large molecular proteins to precipitate fully. The mixture was then centrifuged (4,000 r/min for 10 min), and the supernatant was collected. The concentration of the peptide in the supernatant was the peptide concentration for the subsequent analysis. 0.5 mL of the sample solution was placed in a test tube and diluted to 15.5 mL with distilled water. Then, alkaline copper and Folin phenol reagents (1 mL: 0.5 mL) were added and mixed well. The mixture was incubated at 55 °C for 5 min and then cooled in cold water for 10 min. The OD value at 650 nm was measured using a UV spectrophotometer. The peptide extraction rate in the relevant enzymatic solution was calculated using the following Equation 1:


$$\text{Peptide extraction rate=}\frac{C\times V}{m\times M}\times 100\%$$ (1)

Where *C* is the mass concentration of the polypeptide in the enzymatic solution (mg mL^−1^), *V* represents the volume of the enzymatic solution (mL), *m* is the mass of the *Leucopaxillus giganteus* sample (mg) and, *M* denotes the protein content in *Leucopaxillus giganteus*.

### 2.6 Screening of the optimal ratio of alkaline protease and papain

According to the proportions of alkaline protease and papain specified in Table 1, under conditions of pH 7.2, enzyme addition of 3,000 U/g, and temperature of 50 °C, the most suitable protease ratio can be selected to achieve the best enzymatic digestion effect of *Leucopaxillus giganteus*.

**Table 1:** Protease types and mixing ratios.

Groups	Protease type	Ratio
E1	Alkaline protease:papain	0:1
E2	Alkaline protease:papain	1:0
E3	Alkaline protease:papain	1:1
E4	Alkaline protease:papain	1:2
E5	Alkaline protease:papain	2:1
E6	Alkaline protease:papain	3:4
E7	Alkaline protease:papain	4:3

### 2.7 Single factor experiment

1. Enzyme addition amount: The enzyme digestion temperature is fixed at 50 °C, digestion pH at 7.2, digestion time at 3 h, and enzyme addition controlled at 2000 U/g, 4,000 U/g, 6,000 U/g, 8,000 U/g, 10,000 U/g, comparing the impact of different enzyme amounts on peptide extraction rate.
2. Temperature: With the enzyme addition fixed at 3000 U/g, digestion pH at 7.2, digestion for 3 h, digestion temperature set at 40 °C, 45 °C, 50 °C, 55 °C, 60 °C, comparing the impact of different digestion temperatures on peptide extraction rate.
3. pH: With the enzyme addition fixed at 3000 U/g, digestion temperature at 50 °C, digestion time at 3 h, reaction pH controlled at 6.0, 6.5, 7.0, 7.5, 8.0, comparing the impact of different digestion pH values on peptide extraction rate.
4. Time: The enzyme addition is set at 3000 U/g, digestion pH at 7.2, digestion at 50 °C, digestion time controlled at 2 h, 3 h, 4 h, 5 h, 6 h, comparing the impact of different digestion times on peptide extraction rate.

### 2.8 Plackett-Burman experiment

A Plackett-Burman experiment with *n* = 12 was designed to investigate the significance of factors such as enzyme addition amount, enzymatic hydrolysis time, enzymatic hydrolysis temperature, and enzymatic hydrolysis pH. Each factor was examined separately, as shown in Table 2 for the experimental design, followed by analysis of variance.

**Table 2:** Plackett-Burman test factors and levels.

Factor	Level	
	−1	+1
pH (A)	6.5	7.5
Temperature/°C (B)	45	55
Enzyme addition amount/U/g (C)	4,000	8,000
Time/h (D)	3	5

### 2.9 Box–Behnken response surface optimization experiment

The Box-Behnken (20) design method was employed for response surface optimization to determine the optimal enzyme addition amount, enzymatic hydrolysis pH, and enzymatic hydrolysis temperature. Using the computer software Design-Expert 8.0.6, a 3-factor 3-level design response surface experiment was conducted with peptide extraction rate as the indicator to explore the optimal preparation process for *Leucopaxillus giganteus* peptides. The factor levels for the Box–Behnken response surface experiment are shown in Table 3.

**Table 3:** Box–Behnken response surface test factors and levels.

Level	Factor		
	A pH	B temperature/°C	C Enzyme addition amount/(U/g)
-1	6.5	45	4,000
0	7.0	50	6,000
1	7.5	55	8,000

### 2.10 Antioxidant activity of *Leucopaxillus giganteus* peptides

DPPH free radical scavenging activity: Prepare 0.20 to 1.20 g/L of vitamin C (VC) as the positive control, use anhydrous ethanol as the blank control, prepare the peptide solution obtained in 1.3.1 to 0.50 to 3.00 g/L, accurately transfer different concentrations of samples (VC, peptides) and blank controls 1.00 mL each into test tubes, mix well, then react at room temperature and in the dark for 30 min. Measure the absorbance at a wavelength of 517 nm; calculate the DPPH radical scavenging rate according to the equation:


$$\text{DPPH radical scavenging rate}\;(\%)=(1-\frac{A_{1}-A_{2}}{A_{3}})\times 100\%$$

Where *A_1_* represents the absorbance of the sample; *A_2_* represents the absorbance of DPPH; *A_3_* represents the absorbance of the blank control.

### 2.11 Determination of antibacterial activity of *Leucopaxillus giganteus* peptides

The antibacterial activity of *Leucoagaricus giganteus* peptides was determined via the agar disk diffusion method as previously described with minor modifications (21). Briefly, the bacterial suspension was uniformly spread onto solid agar plates to form a homogeneous bacterial lawn. Sterile filter paper discs (0.5 cm × 0.5 cm) were soaked with 20 μL of *Leucoagaricus giganteus* peptides solution (100 mg L^−1^) and tightly attached to the surface of inoculated agar plates. All treated plates were incubated at 37 °C for 24 h in a constant-temperature incubator. After incubation, the diameters of inhibition zones were measured and recorded to evaluate the antibacterial capacity of the peptide.

### 2.12 Effect of *Leucopaxillus giganteus* peptides on the proliferation of colon cancer Caco-2 cells and liver cancer HepG2 cells

Determination of the anticancer activity of *Leucopaxillus giganteus* peptides using the MTT method (22). Two types of cells were inoculated into 96-well plates at a density of 5 × 103 cells per well. The plates were placed in a 37 °C, 5% CO2 incubator for cultivation. After the cells adhered to the plate, the original culture medium was discarded and replaced with a culture medium containing different concentrations of peptide solutions. A control group and a positive control group (5-fluorouracil, 5 μg mL^−1^) were set up. Each group had 5 replicate wells. After drug administration for 48 h, the supernatant was discarded and 100 μL of MTT culture medium was added for further cultivation for 4 h. The OD value was measured using an enzyme detector at a wavelength of 490 nm. The inhibition rate of the peptide on the proliferation of cancer cells was calculated.

### 2.13 Statistical analysis

Box behnken response surface optimization experiment was conducted using Design Expert 8.0.6. The results were analyzed by one-way ANOVA under the significance level of *p* < 0.05 using SPSS 16.0 software (IBM Corporation, United States). The graphs were drawn by Origin Pro 11 (OriginLab Corporation, United States).

## 3 Results and discussion

### 3.1 Effects of different alkaline protease and papain proportions on the extraction rate of peptides from *Leucopaxillus giganteus*

**Figure 1:**
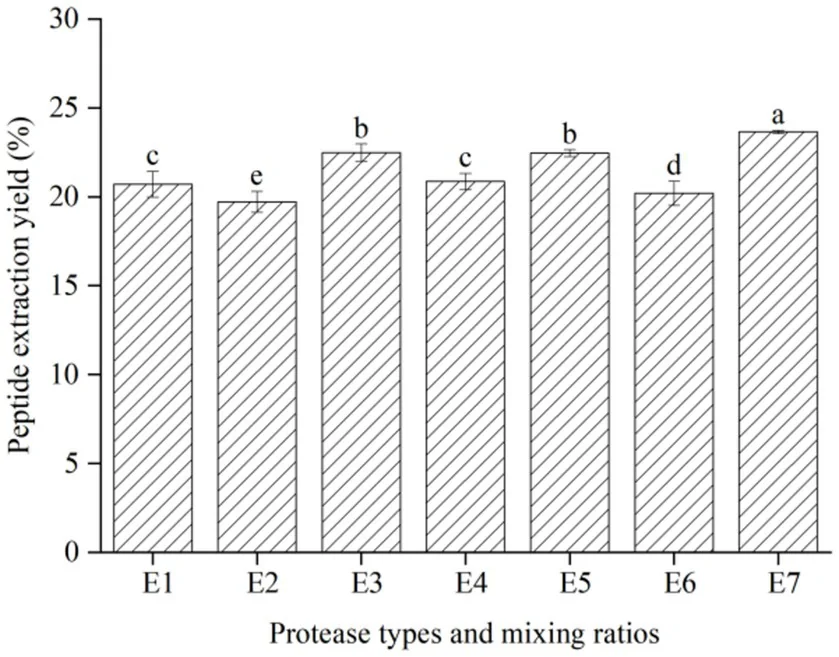
Effects of different ratios of alkaline protease and papain on peptide extraction rate of *Leucopaxillus giganteus* peptides (different lowercase letters above columns indicate significant differences at *p* < 0.05. The same lowercase letters mean no significant difference between groups).

Different proteases possess distinct substrate cleavage specificities, and co-hydrolysis with compound proteases can expand the cleavage range of peptide bonds and synergistically elevate peptide extraction efficiency (23). The sequential action of different proteases to hydrolyze protein substrates is considered one of the most effective methods for obtaining bioactive peptides with specific biological activities (24). As shown in Figure 1, when alkaline protease or papain protease is used alone for peptide extraction, the yield is relatively low, at 19.71 and 20.70%, respectively. When both are used in combination, there is a significant increase in the extraction rate of *Leucopaxillus giganteus* peptides (*p* < 0.05). However, the ratio of alkaline protease to papain protease has a significant impact on the extraction rate of *Leucopaxillus giganteus* peptides. When the ratio of alkaline protease to papain protease is 4:3, the extraction rate of *Leucopaxillus giganteus* peptides is the highest, reaching 23.70%, significantly higher than other ratio combinations (*p* < 0.05). The complementary cleavage sites of alkaline protease and papain enable thorough fragmentation of macromolecular proteins, which fundamentally contributes to the higher peptide yield of two-enzyme combined hydrolysis (25). Therefore, this study selected the ratio of alkaline protease to papain protease as 4:3 for subsequent single-factor enzymatic hydrolysis experiments.

### 3.2 Single factor test

**Figure 2:**
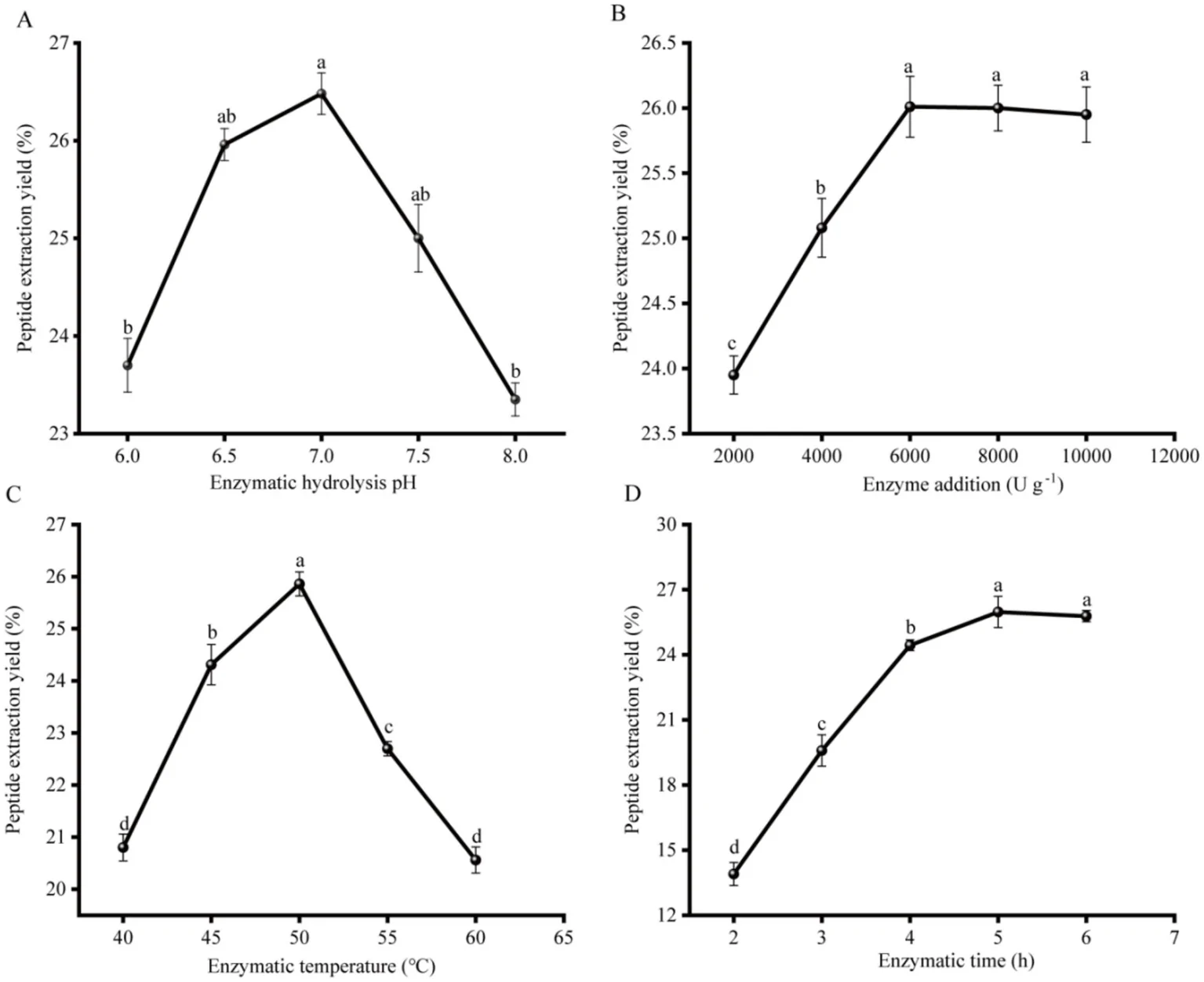
Effects of different factors on the extraction rate of peptides in *Leucopaxillus giganteus*. **(A)** pH, **(B)** Enzyme addition, **(C)** Enzymatic temperature, **(D)** Enzymatic time.

Proteolytic hydrolysis efficiency is strongly governed by the operational parameters of reaction systems. Key variables including pH, temperature, solid–liquid ratio, enzyme addition amount and enzymatic time jointly modulate protease catalytic activity and substrate accessibility, thereby ultimately determining the extent of protein solubilization (26). Under the condition of a 4:3 ratio of alkaline protease to papain protease, the effects of enzymatic pH, enzyme addition amount, enzymatic temperature, and enzymatic time on the extraction rate of *Leucopaxillus giganteu*s peptides were investigated, with results shown in Figure 2.

pH is of crucial importance because each protease has its own optimal pH range. The impact of enzymatic pH on the extraction rate of *Leucopaxillus giganteus* peptides is depicted in Figure 2A. As shown in the figure, the extraction rate of *Leucopaxillus giganteus* peptides initially increases and then decreases with increasing pH. Within the pH range of 6.0–7.0, the extraction rate significantly rises from 23.7 to 26.48% (*p* < 0.05), reaching a peak. However, within the pH range of 7.0–8.0, the extraction rate declines. Enzymes are special proteins, and enzymatic reaction rates are closely related to pH. When pH reaches a certain value, enzyme activity is highest, resulting in the maximum enzymatic reaction rate. Deviations from this optimal pH can affect enzyme stability, inhibit enzyme activity, and even lead to enzyme inactivation (27). Therefore, the optimal enzymatic pH is 7.

The influence of enzyme addition amount on the extraction rate of *Leucopaxillus giganteus* peptides is shown in Figure 2B. The peptide extraction rate gradually increases with the addition of enzymes. At an enzyme addition amount of 6,000 U g^−1^, the peptide extraction rate reaches its peak at (26.01 ± 0.23%), significantly higher than the enzyme amounts of 2000 U g^−1^ (23.95 ± 0.15) and 4,000 U/g (25.08 ± 0.23) (*p* < 0.05). Subsequently, increasing the enzyme addition amount to 8,000 U g^−1^ to 10,000 U g^−1^ does not lead to a significant change in the peptide extraction rate (*p* > 0.05). Insufficient enzyme addition can result in incomplete enzymatic hydrolysis, while excessive enzyme addition can lead to over-hydrolysis. When the enzyme-substrate concentration is saturated, the enzyme reaction rate is at its maximum. Beyond this point, there is a saturation period where the enzyme reaction becomes zero-order. When the enzyme addition exceeds 6,000 U g^−1^, the enzyme-substrate active sites tend to saturate, reducing system flowability and impacting molecular diffusion, leading to a decrease in reaction rate and a more gradual change in peptide extraction.

Temperature is a crucial factor affecting enzymatic hydrolysis efficiency. The impact of enzymatic temperature on the extraction rate of *Leucopaxillus giganteus* peptides is illustrated in Figure 2C. As temperature increases, the extraction rate initially rises and then declines. At 40 °C, the peptide extraction rate is 20.8%, increasing gradually with temperature until reaching the highest extraction rate at 50 °C (25.86 ± 0.23%). Subsequently, within the temperature range of 50–60 °C, the extraction rate significantly decreases (*p* < 0.05). Therefore, the optimal enzymatic temperature is 50 °C. This trend is likely due to incomplete enzyme activation at lower temperatures, leading to lower enzyme activity. As temperature rises, enzyme activity increases, resulting in a higher extraction rate. However, temperatures above the optimal working temperature for enzymes can cause enzyme inactivation, leading to decreased enzyme activity and reduced extraction rates (21).

The impact of enzymatic time on the extraction rate of *Leucopaxillus giganteus* peptides is shown in Figure 2D. With prolonged enzymatic time, the extraction rate of *Leucopaxillus giganteus* peptides increases. At 2 h of enzymatic hydrolysis, the extraction rate is 13.9 ± 0.05%. By 5 h, the extraction rate reaches its peak at 25.86 ± 0.63%, significantly higher than the rates at 3 h (19.59 ± 0.72%) and 4 h (29.44 ± 0.25%) (*p* < 0.05). This is because with shorter extraction times, enzymes and substrates do not fully interact, and peptides are not completely released (28). However, beyond 5 h, specifically at 6 h of enzymatic hydrolysis, the extraction rate does not significantly change (*p* > 0.05). This trend aligns with the results of Gong et al. Therefore, 5 h was the optimal enzymatic hydrolysis duration.

### 3.3 Plackett-Burman test

Table 4 shows the results of the Plackett-Burman experiment, and Table 5 presents the results of the data variance analysis. From Table 5, it can be observed that the model’s significance is extremely significant (*p* < 0.01). The enzymatic pH, enzymatic temperature, and enzyme addition amount have reached a highly significant level for the production of peptides from *Leucopaxillus giganteus* (*p* < 0.01). However, the enzymatic time does not have a significant impact (*p* > 0.05). Therefore, enzymatic pH, enzymatic temperature, and enzyme addition amount were selected for subsequent response surface optimization experiments, with the enzymatic time fixed at 5 h (see Table 6).

**Table 4:** Results of Plackett-Burman test.

Number	Factor				Peptide extraction rate/%
	A	B	C	D	
1	6.5	45	4,000	5	25.32
2	7.5	45	8,000	5	26.39
3	6.5	55	8,000	5	27.15
4	6.5	55	4,000	5	26.63
5	6.5	45	4,000	3	27.35
6	7.5	55	4,000	3	28.14
7	7.5	45	4,000	3	26.79
8	7.5	45	8,000	5	27.12
9	7.5	55	8,000	3	26.83
10	7.5	55	4,000	5	26.67
11	6.5	55	8,000	3	26.45
12	6.5	45	8,000	3	28.14

A expresses enzymatic pH, B expresses enzymatic temperature/°C, C expresses enzyme addition/(U/g), D expresses enzymatic time/h.

**Table 5:** Significant analysis of various factors in Plackett-Burman test.

Source of variance	Freedom	Sum of squares	Mean square sum	F value	*p* value	Significance
Model item	4	1.20	0.26	24.45	< 0.0001	**
A	1	0.38	0.38	15.27	<0.0001	**
B	1	0.62	0.62	56.43	<0.0001	**
C	1	0.22	0.22	10.23	0.0004	**
D	1	0.006	0.006	0.47	0.358	-
Error	6	0.0913	0.0128	-	-	-
Lack of fit	5	0.0913	0.0150	-	-	-
Pure error	1	<0.0001	<0.0001	-	-	-
Total	11	1.2913	-	-	-	-

A expresses enzymatic pH, B expresses enzymatic temperature/°C, C expresses enzyme addition/(U/g), D expresses enzymatic time/h. Decision coefficient = 93.23%, corrected decision coefficient = 89.45%, ** represents extremely significant (*p* < 0.01).

**Table 6:** Results of response surface test.

Number	Factor			Peptide extraction rate/%
	A	B	C	
1	6.5	45	6,000	26.73
2	7.5	45	6,000	28.12
3	6.5	55	6,000	27.6
4	7.5	55	6,000	26.5
5	6.5	50	4,000	28.11
6	7.5	50	4,000	28.18
7	6.5	50	8,000	28.01
8	7.5	50	8,000	28.15
9	7.0	45	4,000	27.9
10	7.0	55	4,000	26.43
11	7.0	45	8,000	26.73
12	7.0	55	8,000	27.4
13	7.0	50	6,000	28.97
14	7.0	50	6,000	28.86
15	7.0	50	6,000	28.9
16	7.0	50	6,000	28.89
17	7.0	50	6,000	28.92

A expresses enzymatic pH, B expresses enzymatic temperature/°C, C expresses enzyme addition/(U/g).

### 3.4 Response surface experiment results

#### 3.4.1 Establishment of response surface model and variance analysis

Performing regression analysis on the experimental data, the regression equation is: Y = 28.91 + 0.063A-0.19B-0.041C-0.62AB + 0.017 AC + 0.53 BC-0.34A2-1.33B2-0.46C2. Conducting variance analysis on the experimental results, the impact of the first-order terms A, B, C, the interaction terms AB, BC, and the quadratic terms A2, B2, C2 on the extraction rate shows statistical significance (*p* < 0.01 or 0.05). Among these, the first-order term B, the quadratic terms AB, BC, as well as A2, B2, C2 have a highly significant impact on peptide extraction rate; the first-order terms A, C have a significant impact, indicating a strong interaction between peptide extraction rate and different factors.

From Table 7, it is evident that the model’s *p*-value is <0.0001, indicating high fitting accuracy of the regression model in this experiment. Therefore, the experimental data can be well explained through the multiple regression model, showing that factors such as pH, enzymatic temperature, and enzyme addition amount have a significant impact on peptide extraction. The lack-of-fit *p*-value is 0.7962, which is not significant, implying model stability, good fitting effect, no apparent nonlinear effects or interactions, and reliable experimental data. Table 8 shows that the model’s correlation coefficient is close to 1, with a small difference between the adjusted and predicted correlation coefficients (<0.2), indicating good predictive capability of the model. With a coefficient of variation less than 10%, the model’s reliability is high, further validating the regression model’s reliability.

**Table 7:** Results of variance analysis of regression model.

Source of variance	Sum of squares	Freedom	Mean square	F value	*p* value	Significance
Model item	12.52	9	1.39	1158.77	< 0.0001	**
A	0.031	1	0.031	26.02	0.0014	*
B	0.30	1	0.30	250.11	< 0.0001	**
C	0.014	1	0.014	11.34	0.0120	*
AB	1.55	1	1.55	1290.92	< 0.0001	**
AC	1.23E-03	1	1.23E-003	1.02	0.3461	
BC	1.14	1	1.14	953.52	< 0.0001	**
A^2^	0.48	1	0.48	397.07	< 0.0001	**
B^2^	7.49	1	7.49	6240.34	< 0.0001	**
C^2^	0.89	1	0.89	738.79	< 0.0001	**
Residual error	8.41E-03	7	1.201E-003	-	-	-
Misfit term	1.725E-003	3	5.75E-04	0.34	0.7962	-
Pure error	6.680E-003	4	1.67E-03	-	-	-
Total error	12.53	16	-	-	-	-

A expresses enzymatic pH, B expresses enzymatic temperature/°C, C expresses enzyme addition/(U/g). ** indicates extremely significant (*p* < 0.01), *indicates significant (*p <* 0.05).

**Table 8:** Results of error analysis of regression model.

Statistical items	Statistical value	Statistical items	Statistic
Standard deviation	0.035	Correlation coefficient	0.9993
Average value	27.91	Correction correlation coefficient	0.9985
Coefficient of variation (%)	0.12	Prediction correlation coefficient	0.9970
Residual prediction sum of squares	0.038	Precision	93.335

In conclusion, the peptide extraction rate regression model fits well, allowing optimization of the extraction process for *Leucopaxillus giganteus* peptides to enhance the extraction rate. Additionally, optimization experiment results indicate the degree of influence of each factor on *Leucopaxillus giganteus* peptide extraction rate in the following order: B > A > C, meaning enzymatic temperature has the greatest impact, followed by enzymatic pH, with enzyme addition amount having the least impact. This suggests that when optimizing peptide extraction conditions, enzymatic temperature should be given priority.

#### 3.4.2 Establishment of response surface model and variance analysis

**Figure 3:**
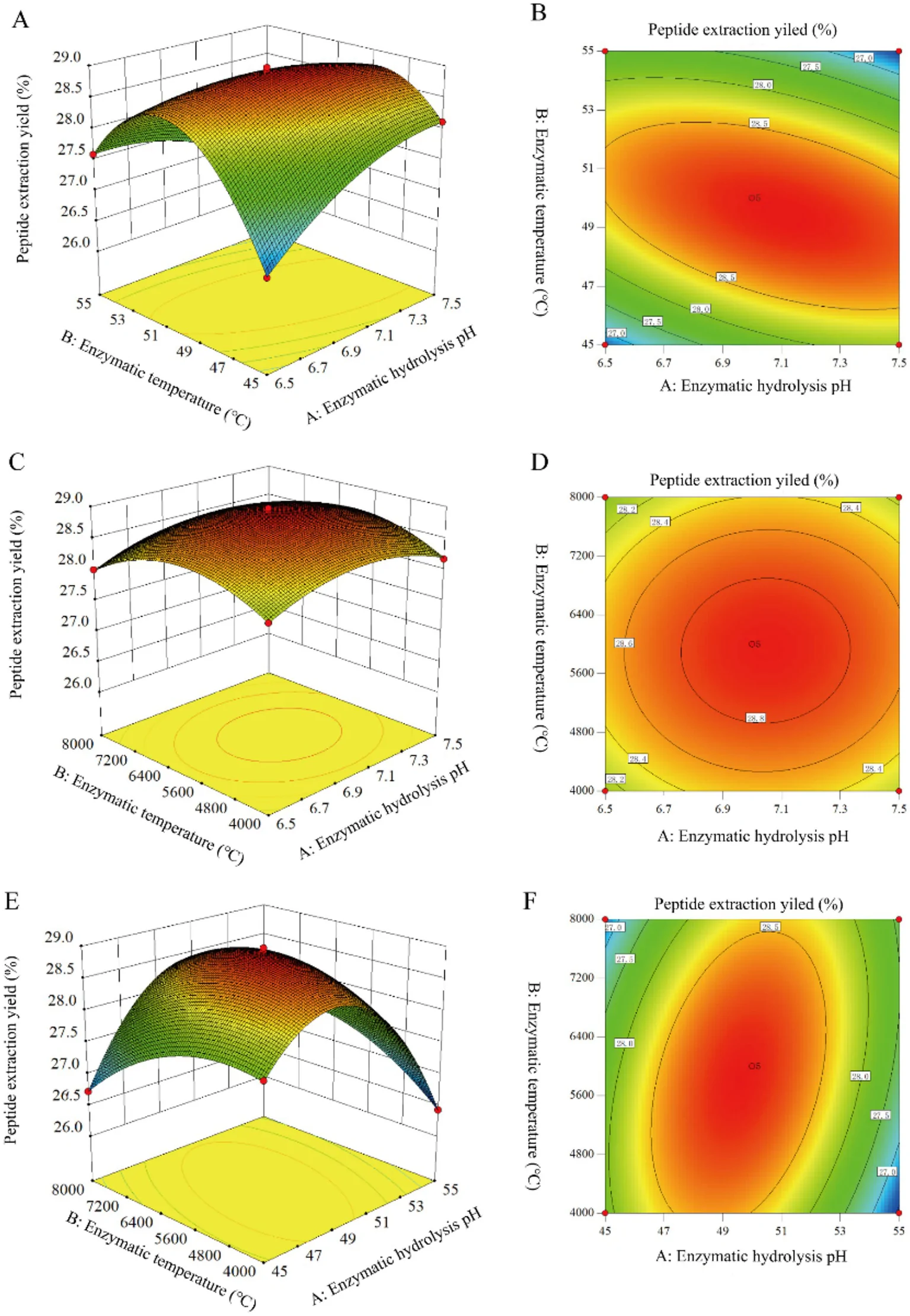
Response surface diagram of interaction between two factors: **(A)** Response surface plot for the effect of enzymatic pH and enzymatic temperature on peptide extraction rate; **(B)** Contour plot for the effect of enzymatic pH and enzymatic temperature on peptide extraction rate; **(C)** Response surface plot for the effect of enzymatic pH and enzyme addition amount on peptide extraction rate; **(D)** Contour plot for the effect of enzymatic pH and enzyme addition amount on peptide extraction rate; **(E)** Response surface plot for the effect of enzymatic temperature and enzyme addition amount on peptide extraction rate; **(F)** Response surface plot for the effect of enzymatic Contour plot for the effect of temperature and amount of enzyme added on peptide extraction rate.

Three-dimensional plots and contour maps can intuitively demonstrate the effects and interactions of multiple independent variables on the dependent variable, as shown in Figure 3. The three-dimensional plot reveals a complex relationship between the extraction rate of peptides from *Leucopaxillus giganteus* and the three factors. The impact of different factors on peptide extraction rate is not linear but rather forms a parabolic surface. The downward opening of the parabolic surface indicates a maximum value, suggesting the presence of optimal enzymatic temperature, pH, and enzyme addition amount for achieving the highest peptide yield. Furthermore, there are interactions among the three factors, as indicated by the elliptical shape of the contour map, showing that the factors do not act independently but influence each other.

Combining Figure 3 and Table 7 reveals that the response surface curve for the interaction between enzymatic temperature and enzymatic pH is steepest, with closely spaced contour lines, indicating a steep effect surface curve. Therefore, the impact of interactions on peptide extraction rate from *Leucopaxillus giganteus* is as follows: enzymatic temperature and enzymatic pH > enzymatic temperature and enzyme addition amount > enzyme addition amount and enzymatic pH.

#### 3.4.3 Verification of box Behnken response surface model

Through the analysis of the response surface model using Design-Expert 8.0.6.1 software, it was determined that the optimal process parameters for preparing peptides from *Leucopaxillus giganteus* are: enzymatic temperature of 49 °C, enzyme addition amount of 5,870 U/g, and enzymatic pH of 7.1, resulting in a theoretical peptide extraction rate of 29% from *Leucopaxillus giganteus* under these conditions. Three validation experiments were conducted based on the modified optimal conditions, resulting in a peptide extraction rate from *Leucopaxillus giganteus* of 28.93%, very close to the predicted value. This indicates the feasibility of optimizing the extraction process using the response surface method.

### 3.5 The antioxidant activity of peptides from *Leucopaxillus giganteus*

**Figure 4:**
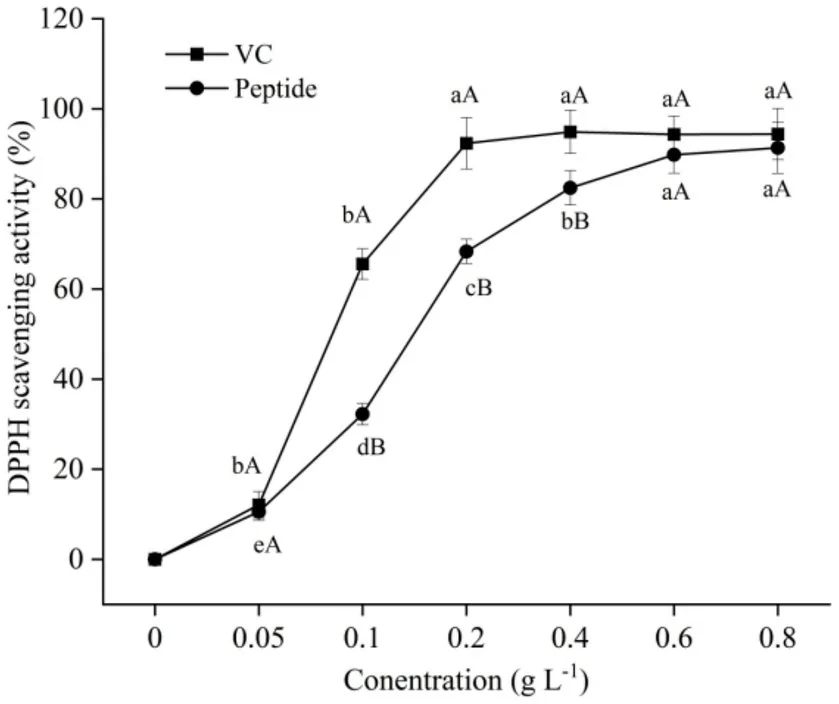
DPPH scavenging activity of peptides from *Leucopaxillus giganteus*.

The common method for evaluating the antioxidant properties of bioactive peptides is to measure their ability to scavenge DPPH radicals (29). Figure 4 illustrates the *in vitro* antioxidant activity of *Leucopaxillus giganteus* by assessing its ability to scavenge DPPH free radicals. Within the range of 0 to 0.8 g/L, the scavenging effect of *Leucopaxillus giganteus* peptides and VC on DPPH free radicals shows an increasing trend. From 0.05 to 0.8 g L^−1^, the DPPH free radical scavenging rate significantly increases from 10.55 to 91.35% (*p* < 0.05), demonstrating a dose-dependent relationship. The scavenging effect of *Leucopaxillus giganteus* peptides on DPPH free radicals enhances with increasing concentration. Despite a certain difference in the scavenging effect of free radicals between peptides and VC, *Leucopaxillus giganteus* peptides exhibit a significant scavenging effect on DPPH free radicals (*p* < 0.05), indicating their strong antioxidant activity.

### 3.6 Antibacterial activity of peptides from *Leucopaxillus giganteus*

**Figure 5:**
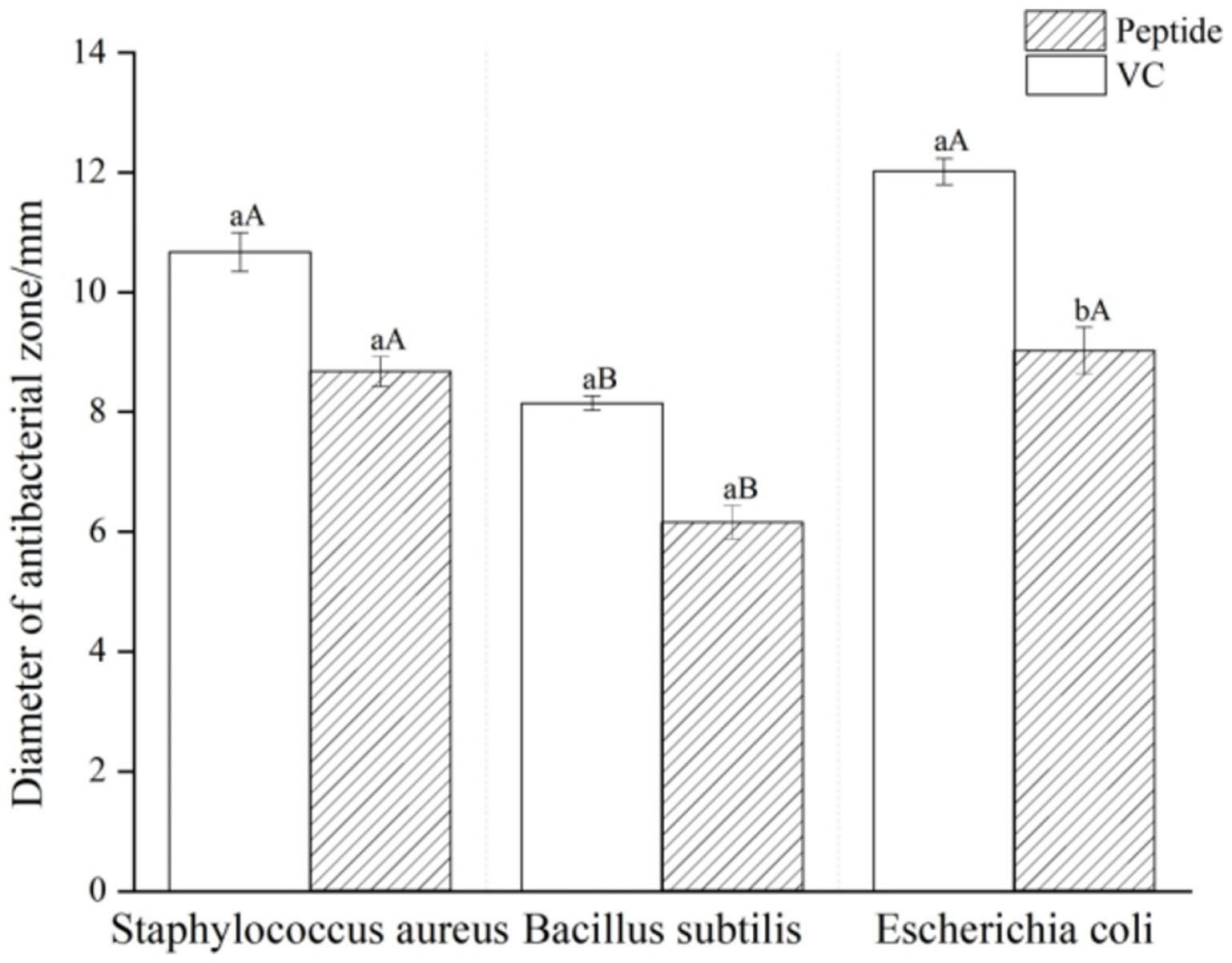
Antibacterial effects of peptides in *Leucopaxillus giganteus*.

From Figure 5, it can be seen that the inhibitory effect of *Leucopaxillus giganteus* peptides on *Bacillus subtilis* and *Staphylococcus aureus* is not significantly different from that of VC (*p* > 0.05). It is speculated that the antibacterial effect is due to the rich amino acids in the peptides, which can bind to specific substances on the bacterial cell membrane, disrupt the cell structure, and inhibit the germination of bacterial spores, thereby exerting an antibacterial effect. On the other hand, the inhibitory effect of *Leucopaxillus giganteus* peptides on *Escherichia coli* is weaker, with an inhibition zone diameter of 9.02 mm, significantly lower than VC (12.02 mm) (*p* < 0.05). This may be related to the structural characteristics of *Escherichia coli* itself. Therefore, the *Leucopaxillus giganteus* peptides prepared in this experiment have a good inhibitory effect on *Escherichia coli*, *Staphylococcus aureus*, and *Bacillus subtilis*.

### 3.7 The anti-cancer activity of peptides from the *Leucopaxillus giganteus*

**Figure 6:**
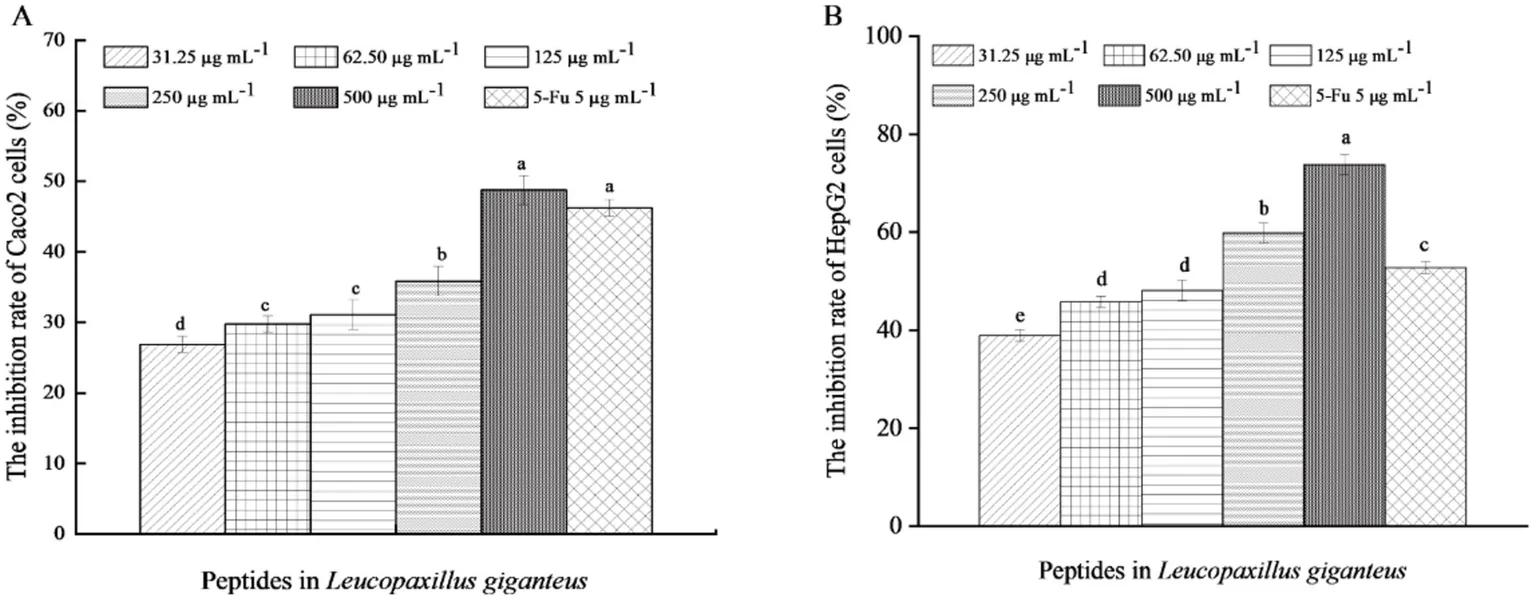
The peptides of *Leucopaxillus giganteus* inhibit the proliferation of colorectal cancer Caco2 cells **(A)** and liver cancer HepG2 cells **(B)**.

Malignant tumors have become a major worldwide public health crisis, causing massive mortality each year (30). Compared with traditional anti-cancer small-molecule drugs, tumor-inhibiting bioactive peptides and proteins exhibit unique merits: strong cell permeation, precise recognition of tumor-specific signaling pathways, and freedom from genotoxic side effects (31). As shown in Figure 6, the inhibitory effect of *Leucopaxillus giganteus* peptides on colorectal cancer Caco2 cells and liver cancer HepG2 cells is shown. As seen in Figure 6A, different mass concentrations of *Leucopaxillus giganteus* peptides have a significant inhibitory effect on Caco2 cells (*p* < 0.05). With the increase in mass concentration of *Leucopaxillus giganteus* peptides, the proliferation inhibition rate of Caco2 cells gradually increases, with an IC_50_ of 0.54 μg mL^−1^. The inhibitory effect of different mass concentrations of *Leucopaxillus giganteus* peptides on liver cancer HepG2 cells is depicted in Figure 6B. The results indicate that as the mass concentration of *Leucopaxillus giganteus* peptides increases, the proliferation inhibition rate of HepG2 cells significantly increases (*p* < 0.05), with an IC50 of 0.15 μg mL^−1^.

It appears that both *Leucopaxillus giganteus* peptides and the positive control group can effectively inhibit the growth of tumor cells. Furthermore, their inhibitory effects on different tumor cell lines vary. In this experiment, *Leucopaxillus giganteus* peptides exhibit very good inhibitory effects on colorectal cancer Caco2 cells and liver cancer HepG2 cells. This may be attributed to the relatively small molecular weight of *Leucopaxillus giganteus* peptides, which can regulate biological processes such as cell division and apoptosis inhibition upon entering cancer cells (32). Therefore, as small molecules, *Leucopaxillus giganteus* peptides can enter cancer cells, disrupt their normal structure and function, and ultimately induce cancer cell death.

## 4 Conclusion

The protein content of *Leucopaxillus giganteus* is rich. In this study, using *Leucopaxillus giganteus* as the experimental material and peptide extraction rate as the index, the peptide preparation process was optimized. After optimization, the peptide extraction rate can reach 28.93%. The peptides prepared under this process exhibit good inhibitory effects on *Escherichia coli*, *Bacillus subtilis*, and *Staphylococcus aureus*. Additionally, they show good proliferation inhibition on colorectal cancer Caco2 cells and liver cancer HepG2 cells. The conclusion of this study indicates that the fruiting bodies of *Leucopaxillus giganteus* have potential pharmaceutical applications and can serve as a reference for further large-scale development of *Leucopaxillus giganteus* resources. The current research has only conducted preliminary *in vitro* antibacterial and anti-tumor cell tests. Future studies need to further carry out comprehensive *in vivo* pharmacodynamic and toxicity research to provide more sufficient theoretical support for the functional development and application of this substance.

## Data Availability

The original contributions presented in the study are included in the article/supplementary material, further inquiries can be directed to the corresponding author.
